# Bioprosthetic Aortic Valve Replacement in <50 Years Old Patients – Where is the Evidence?

**DOI:** 10.21470/1678-9741-2018-0374

**Published:** 2019

**Authors:** Amer Harky, Michael Man Yuen Suen, Chris Ho Ming Wong, Abdul Rahman Maaliki, Mohamad Bashir

**Affiliations:** 1 Department of Cardiothoracic Surgery, Liverpool Heart and Chest Hospital, Liverpool, UK; 2 Faculty of Medicine, The Chinese University of Hong Kong, Hong Kong.; 3 Manchester Royal Infirmary, Oxford Road, Manchester, UK.

**Keywords:** Aortic Valve Stenosis – Etiology, Young Adult, Transcatheter Aortic Valve Replacement – Methods

## Abstract

Aortic valve disease is one of the most common valvular heart diseases in the cardiovascular category. Surgical replacement of the diseased aortic valve remains the definitive intervention for most diseases.

There is a clear consensus that in young patients who require aortic valve replacement, a mechanical prosthesis is the preferred choice due to its durable prosthesis without fear of wear and tear over time. However, this comes at the expense of increased risk of bleeding and thromboembolic events; in addition, there is a lack of strict evidence in using bioprosthesis in patients younger than 50 years. The objective of this review article is to assess the current evidence behind using bioprosthetic aortic valve in this young cohort.

**Table:** 

Abbreviations, acronyms & symbols				
AHA/ACC	= American Heart Association and the American College of Cardiology		INR	= International normalized ratio
			LVMI	= Left ventricular mass index
AS	= Aortic stenosis		NYHA	= New York Heart Association
BAV	= Bicuspid aortic valve		PARTNER	= Placement of Aortic Transcatheter Valve
CABG	= Coronary artery bypass grafting		PCI	= Percutaneous coronary intervention
CAD	= Coronary artery disease		PPM	= Patient-prosthesis mismatch
CPB	= Cardiopulmonary bypass		SAVR	= Surgical aortic valve replacement
CEP	= Carpentier-Edwards Perimount		SVD	= Structural valve deterioration
EPP	= Edwards Prima Plus		TAVR	= Transcatheter aortic valve replacement
ESC	= European Society of Cardiology		VA	= Veterans Affairs

## INTRODUCTION

Cardiovascular diseases are still among the most important disease entities in the world affecting different countries, from developing to developed places. Valvular pathologies exert effects on patients of all ages. From children born with valvular congenital abnormalities, adults affected by rheumatic heart fever to elderly developing atherosclerosis, heart valves are never spared from pathologies. Annually, being the definitive treatment of valvular diseases, 300,000 heart valve surgeries are performed worldwide^[1]^. Among the valvular pathologies, aortic stenosis is the most widely encountered in Western countries^[2]^. Studies have found that more than one in eight people over 75 years of age have moderate or severe aortic stenosis^[3]^, awaiting surgical correction, not counting asymptomatic patients, often younger in age.

Aortic stenosis in the young is often easily overlooked compared to the elderly. As a result of stenotic bicuspid aortic valve^[4]^. this congenital anomaly happens in as many as 1% of the total population and is the second most common aetiology of aortic stenosis^[5]^. Despite evidence supporting pharmacological management, valve replacement is the only definitive treatment to date. This review will investigate aortic stenosis interventions with focus on the options available to the younger populations and developments in related modalities, provide evidence in support of these treatments, and highlight a few unanswered questions in related fields.

### Natural History of Aortic Stenosis

Aortic stenosis (AS) results from three main causes: 1) calcification of a normal trileaflet valve; 2) calcification of a congenitally abnormal bicuspid or unicuspid valve; and 3) rheumatic heart valve disease. Other metabolic conditions or rare diseases, such as systemic lupus erythematosus or mineral metabolism problems, can contribute to the development of AS.

In the elderly, calcific AS often progresses from aortic sclerosis, aortic valve thickening involving lipid accumulation and calcium deposition, involving processes that are also found in atherosclerosis. Progression to AS is defined by antegrade velocity across a valve above 2m/sec, resulting from outlet narrowing. This process is often compensated hemodynamically, and patients therefore remain asymptomatic for long time.

Aortic stenosis in younger patients is often a result of bicuspid aortic valve (BAV)^[6]^, although rheumatic heart disease is also possible. In BAV, valve sclerosis begins when patients are in their twenties. It is known that bicuspid is more prone to stenosis progression compared to tricuspid normal valves^[7]^. Calcium deposition sets during the fourth decade of life. Progression to AS depends on age. Over 70% of patients with bicuspid aortic valve have AS by the age of 70. Concerning the long-term survival of AS patients, presence of symptoms is an important indicator of prognosis. Survival for symptomatic AS is 2-5 years^[8]^. without intervention. Sudden death, although rare, with odds of less than 1% per year, can also take place^[6]^. As a result, intervention is required.

### Evidence of Surgical Aortic Valve Replacement

Surgical aortic valve replacement (SAVR) has been the gold standard of the definitive treatment for decades^[9]^. Despite being a complex procedure, and that patients are often presented with other morbidities, hence suffering from greater perioperative risks, it has been constantly developed with efforts being put into surgical methods and postoperative care, resulting in low mortality in the short and long term and reduction of complications. As early as in 1996, Bessel et al.^[9]^ conducted a single institution prospective study on 1,322 patients undergoing aortic valve replacement surgery, mostly with mechanical prosthesis. It showed that the hospital mortality rate was merely 3.3%^[9]^. Throughout the years, risk profiles of patients undergoing SAVR have worsened. A Scottish retrospective study consisting of 4,124 patients undergoing primary aortic valve replacement from 1996 to 2011 showed that patients with diabetes rose from 1.9% in 1996 to 12.6% in 2011; those with hypertension from 26.4% to 56.1%; and those with cerebrovascular diseases from 3.7% to 9.8%^[10]^. However, perioperative death maintained at similar level to 3.1%.

As for the efficacy of surgical valve replacement, it is summarized in a systematic review carried out in 2004 by Sharma et al.^[11]^. With left ventricular hypertrophy regression and restoration of left ventricular ejection fraction being the markers long term outcomes of surgery, patients with AS had significant functional improvement after valve replacement. Patients with low EF preoperatively improve significantly (from 28% to 40%) at 6 to 41 months of follow-up^[11]^. Left ventricular mass index (LVMI) also fall significantly, evident within 6 months of surgery. LVMI falls from 181 g/m^2^ to 124^[11]^.

It can be concluded from the aforementioned evidence that SAVR is an effective and relatively safe intervention of AS.

### Choice of Valve: A Changing Landscape

The choice of valve replacement has been a question of constant debate. Conventionally, it is a tug of war between mechanical and biological, otherwise known as bioprosthetic valve. Despite the recommendations of various guidelines regarding use of certain types in different patient groups, the fact that these guidelines are still changing highlights that this debate is not settled.

### Types of Valves

Prosthetic heart valves have had a history of almost 60 years. Starr-Edwards ball mechanical heart valves have been implanted from the 1960s and remained the valve of choice for 20 years. Despite popularity, it has poor hemodynamics. Its dominance was taken over by bileaflet valves such as the St. Jude^®^ mechanical valve introduced in 1970s, which still remains the valve of choice. More than 600,000 were implanted^[12]^. There are also disc valves such as the Bjork-Shiley and the Medtronic-Hall prosthesis, which have better hemodynamic performance than the ball valve, but are prone to disastrous thrombosis in case of valve fracture and are less commonly used nowadays^[13]^.

Bioprosthetic valves are divided into 1) homograft or allograft, 2) xenograft, 3) stentless valve. Homografts are derived from cadavers. Supply as an implant for aortic valve replacement is often limited. Allograft comes from the patient’s own pulmonary graft and manipulated as aortic valve. Evidence-based usage is limited to paediatric patients. Xenograft includes bovine aortic valve and porcine pericardial valve. Bovine valve is attached to a stent and is susceptible to long-term prosthesis stenosis. Stentless porcine valves are obtained by removing the porcine aortic valve along with nearby aorta and is less prone to stenosis with better hemodynamics^[14]^. With constant trials and research, bioprosthetic valves now come with improved durability and performance of bioprosthetic valves, through new valve designs, anticalcification treatment, valve materials and more. Generally speaking, mechanical valves are more durable and free of deformation but requires lifelong anticoagulation and comes with risk of endocarditis. Risks of thromboembolism in mechanical valves depend on the type of valve used^[15]^. In the bioprosthetic valve, lifetime anticoagulation is avoided, unless indicated in other conditions. Disadvantages of bioprosthesis includes susceptibility to structural valve deterioration (SVD).

As to the trend of prosthesis choice, usage of bioprosthesis is on a rising trend. According to Issacs et al.^[16]^, in the USA bioprosthesis only made up to 37.7% of aortic valve implants between 1998 and 2001. The number rose to 63.3% between 2007 and 211.

### Younger Population: The Scope of Debate

The choice of aortic valve implant is overseen by guidelines determined by various factors. Far from a definitive cut-off, the age of implantation of bioprosthetic heart valves has been lowering as they are gaining popularity. Currently, bioprosthesis is more preferred in the elderly patient, while mechanical prosthesis in the young; the median age of bioprosthesis recipients is 74, while the mean age of mechanical prosthesis recipients is 67^[16]^.

The recommended age for implanting bioprosthetic valves has dramatically decreased in recent years. In 2007, the European Society of Cardiology (ESC) recommended the use of bioprosthetic valve for patients older than 65 years of age, which was subsequently lowered to 60 years in 2012^[17]^. The guidelines of the American Heart Association and the American College of Cardiology (AHA/ACC) suggested that the bioprosthetic valve be considered to implant over the age of 65 in 1998 and 2006, respectively. The recommended age was lowered to 60 in 2010. A recent update in 2017 recommended that the age limit be lowered to 50 years of age^[18]^. With the continuous improvement in the durability of valves and lowered risk of reoperation^[19]^, increasing evidence suggests that such valves may be suitable for younger patients than the suggestion of current guidelines, that is, under 50 years, have been reported.

### Support of Mechanical Prosthesis in The Young

Traditionally, it is understood that the mechanical valve is associated with fewer incidences of valve failure and lower rate of reoperation compared to the biological valve, and is implanted in younger patients for its long-term reliability. A 25-year follow-up of patients receiving St. Jude® mechanical valve, 81% ± 10% of them are free of reoperation, 52% ± 8% of thromboembolism, 64% ± 6% free of bleeding and 97% ± 1% free of endocarditis. No structural failures of the prosthesis are reported^[20]^. Mechanical valves had been the prostheses of choice for a long time.

Concerning the valve replacement choice in younger adults, with longer life expectancy and higher accumulated risk of suffering from prosthesis complications, evidence in the past has shown that mechanical valves have a better performance in these groups of patients over bioprosthetic valves. In terms of outcomes of the surgery, Bech-Hanssen et al.^[21]^ claimed that left ventricular mass regression was higher in mechanical than biological valves two years after the operation. Mechanical valves reduce 21.7 g more than biological valves when the prosthesis size was taken into account. The Doppler gradient was also 4 mmHg lower in the mechanical valve than in the same size bioprosthesis.

In 2000, 15 years after the Veterans Affairs (VA) randomized trial, Hammermeister et al.^[22]^ concluded that the bioprosthetic valve does not bring lower valve-related complications. Embolism rate was identical for both mechanical and bioprosthetic group (18%) in their study in which 394 patients receiving either type of valve was followed up for 15 years. Complications were due to risk factors related to patients, such as thromboembolic risks^[22]^.

In terms of overall mortality, Brown et al.^[23]^ compares the outcomes of 50- to 70-year-old patients who received aortic valve replacement, either St. Jude® mechanical or Carpentier-Edwards^®^ bioprosthesis. They found that the 10-year survival of patients with mechanical valves is 68%, *versus* 50% in bioprostheses. Only 2% of mechanical valves recipients needed to be reoperated within 10 years, while 10% of those receiving bioprostheses required reintervention. Recalling that 19% of patients with bioprosthesis were taking warfarin, the authors also argued that the use of bioprostheses does not necessarily mean that patients can be free from anticoagulants if they have other related diseases. Therefore, they were not free from adverse complications of anticoagulation.

### Evidence of Bioprosthesis Implantation in Younger Patients

#### Need of Consideration

Statically speaking, bioprosthesis is not a valve of choice for young people regarding the physicians’ perspectives. Issacs et al.^[16]^ reported that, for patients aged 18 to 54, only 27% of the implants are bioprostheses and the remainder, mechanical. In contrast, as the bioprosthesis is in the development trend, there are increasing evidence suggesting a reduction in the age threshold for the use of a bioprosthetic valve, meaning that younger patients should be considered in the choice of type of prosthesis. This is corroborated by the long-term durability of bioprosthetic valves.

In the past, a major claimed disadvantage of the bioprosthetic valve was its lower durability, hence prone to valve deterioration or reoperation. However, the long-term follow-up of patients receiving bioprosthetic implants have revealed otherwise. Johnston et al.^[24]^ followed 12,569 patients who received the Carpentier-Edwards Perimount (CEP) aortic valves for 20 years. Results published in 2015 show that valve explant due to SVD is rare (15% overall). Valves have good durability also in younger patients, of whom 55% are spared from explants after 20 years. CEP valve durability in younger patients is also supported in a cohort study following patients under 60 years using CEP bioprosthesis for up to 20 years^[25]^. It reported a low incidence of SVD, standing at only 37.2% ± 5.4% and with expected valve durability of 17.6 years.

Meanwhile, Edwards Prima Plus (EPP) Stentless Bioprosthesis also demonstrated good results, as suggested by Christ et al.^[26]^. In their study, 120 patients with a median age of 53.1 received replacement with EPP valve and were followed for up to 14 years. Actuarial survival stands at 69.5% ± 5.5% at 10 years. Comparatively, the VA trial following patients of median age of 59 years receiving mechanical prosthesis only achieves survival rate of 47% in 10 years^[22]^. Concerning effect of valve deterioration in EPP valve, freedom from reoperation reaches 85.6% ± 3.7% at 10 years^[26]^.

The Medtronic Mosaic^®^ porcine valve is another 3^rd^ generation bioprosthesis with long-term follow-up data available. Anselmi et al.^[27]^ conducted a retrospective cohort and analyzed the long-term durability of this valve for up to 15 years. Among the age group below 70 years old, the actuarial rate of freedom from SVD is 75.5% and the freedom of reoperation from SVD reaches 86.1%, at 15 years. It was also shown that the Mosaic prosthesis demonstrates a very low risk of endocarditis – the freedom from prosthesis endocarditis ranges from 97.6% to 98.9% among age groups and does not show statistical difference. No adverse prosthesis complications are age-dependent. That means younger patients implanted with the valve will not face higher risk of complications^[27]^.

Second-generation aortic valve prostheses have also demonstrated excellent durability. Yankah et al.^[28]^ evaluated the Mitroflow pericardial prosthesis by following up 1,513 patients over 21 years. Freedom from reoperation due to SVD was 88.6%. Anticoagulation could be withdrawn from 92% and antiplatelet agent from 90% of patients, after postoperative administration. Actuarial freedom from valve-related death was 83% at 20 years^[28]^.

Conclusions from various studies on CEP, EPP and Mitroflow valves appear congruent. Bioprosthetic valves used in SAVR have excellent durability. It gives rise to the question whether the age threshold of patients receiving bioprosthesis should be further reduced.

#### Present Evidence in Favour of Bioprosthesis in the Young

A meta-analysis by Lund and Martin published in 2006 compared the risk-adjusted mortality rate between bioprosthetic valve and mechanical valves in aortic valve replacement. This study included 32 articles (15 on mechanical valves and 23 on bioprosthetic valves), a total of 17,439 patients and 101,819 patient-years. Interestingly, the mean age of patients receiving mechanical implants is 58.0 while bioprosthesis is 68.8. It gives rise to doubt whether poorer outcomes in bioprosthesis recorded in some studies were contributed by the greater age and more comorbidities in recipients. After correction for age and significant risk factors, such as NYHA III and IV and CABG, no difference in mortality was found between the two types of valves, regardless of the patients’ age^[29]^. Therefore, we can see that age should not be the only consideration in selecting the right type of valve for patients.

In another single-center study, patients (≤60), mean age 53.1, who underwent stentless aortic valve replacement between 1993 to 2001 were followed up for 17 years. Patients were divided into below and above the age of 50 years. Comparing the two groups, Christ et al. found that age was not a significant factor for survival and freedom from reoperation^[26]^. The following items may provide some insight on how age and other factors affect the choice of prosthesis from different angles.

#### Long-Term Survival

One of the main focuses of the argument is the long-term survival of patients receiving the valve implants. In the Veterans Administration (VA) study, patients with mechanical valves had lower mortality rate (66%) compared to the bioprosthetic valve (79%) at 15 years^[22]^. The Edinburgh heart valve study reported a survival advantage of mechanical valves over 12 years, but after 20 years there was no difference between the two valves^[30]^.

More effort was developed to compare the long-term outcome of two valves amidst continual development of bioprostheses that might deliver better results. A more recent randomised trial has been carried out by Stassano et al.^[31]^ to determine compare the long-term mortality of patients receiving biological and mechanical valve, especially within a younger population. A total of 310 patients aged 55 to 70 were included, and the St Jude Medical^®^ and CarboMedics^®^ mechanical valves were compared to the second generation Carpentier-Edwards valve. The study could not demonstrate a statistically significant difference in the 13-year survival rate between the two valves: the mortality in patients with bioprosthesis was 30.6% and in patients with mechanical prosthesis was 27.5%.

Difficulties were found in the evaluation of the long-term outcome of bioprosthesis in younger population. There are much fewer young patients requiring aortic valve implants compared to the older groups. They have a more diversified background and comorbidities.

Despite this, Ruel et al.^[32]^ attempted to analyse long-term outcomes of patients aged 18-50 who received aortic valve replacement. In their study, 309 patients received isolated aortic valve replacement and were followed up for 15 years^[32]^. The survival rate between mechanical and biological valves shows no difference, regardless the reoperation rate or valve deformation. Survival rate of patients with mechanical prosthesis is 78.9% at 15 years, and with bioprosthesis is 79.2%. Meanwhile, Ruel suggested that bleeding events, both intracranial and extracranial, occur at a higher chance in patients with mechanical prosthesis, in conjunction with the finding that all bleeding events occur in patients using warfarin, except for two cases. Embolic stroke as a complication has no significant difference between the two prostheses.

#### Factors to Long-Term Survival after Valvular Implant

Various results seem to have demonstrated that long-term survival is not affected by the prosthesis type. In search of the other factors that contribute to the long-term mortality of patients receiving the operation, patient’s pre-morbidities and risk profiles are found to contribute much more^[33]^. An example can be demonstrated by the cohort conducted by Brown et al.^[23]^. Despite an advantage of mechanical over bioprosthetic valve is reported, the fact that clinicians preferentially offer bioprosthesis to patients with more pre-morbidities affects the patient outcome. Preoperatively, bioprosthetic valve patients had greater proportion of NYHA III or IV (72.7% *vs*. 62.9%) than mechanical valve patients. Rates of renal failure (5.5% *vs*. 4.6%) and diabetes (17.3% *vs*. 15.0%) are also higher in the bioprosthesis group^[23]^. Whether it is the valve type affecting the patient outcome or the patient’s impression of pre-morbidities dictating the clinicians' choice remains a question.

### Reoperation and its Risk

Another factor that numerous studies believed to affect patient outcome is the reoperation rate following the first valve replacement. However, the mere incidence of reoperation should not be considered a complication or failure of the primary operation. Stassano et al.^[31]^ report a substantial difference in reoperation rate between bioprosthetic and mechanical valve groups in the long-term (2.32% per patient-year *vs*. 0.62%), but at the same time a similar long-term survival rate. Ruel et al.^[34]^ reported a similar pattern. It is clear that the difference in reoperation rate does not directly add up to mortality. Instead, reoperation should be defined as part of the treatment of aortic valve replacement with bioprosthesis in the long run^[34]^, as early as when patients are offered primary surgery. How patients are managed perioperatively, how anticoagulation is prescribed and how infection is prevented are what affecting mortality. In fact, the risk of reoperation is no greater than primary surgery thanks to advancements in surgical techniques^[33]^.

In contrast, mechanical prosthesis is not free from reoperation despite the absence of SVD. Edinburgh trial reports that 7.3% of mechanical valves require reoperation at 20 years; VA study reports an incidence of 10% at 15 years^[22,30]^. However, percutaneous valve-in-valve procedure provides an option in case of prosthesis failure instead of warranting reoperation, which is not applicable to pre-existing mechanical prostheses. These are the various factors that should be addressed altogether when considering the likelihood of reoperation.

#### Prosthesis Complications

In addition to the long-term mortality, late complications of the prosthesis should be compared. Mechanical prosthesis is more likely to cause complications that translate into profound morbidity. Stassano et al.^[31]^ reported odds of bleeding in mechanical prosthesis at 1.47% per patient-year, against 0.72% of bioprosthesis. Thromboembolism rate is 0.54% per patient-year for mechanical valve, 0.24% for bioprosthesis. Chikwe et al.^[33]^ reviewed various studies, finding that the annual rate of thromboembolism and bleeding is 3% and 2.8%, respectively, for the mechanical valve, higher than 2.5% and 1.5% for the bioprosthetic valve. These complications can be devastating and irreversible. They are also acute in onset and deprive the patients’ role in deciding a management direction. Whereas SVD, more likely a complication of bioprosthetic valve, is less acute in presentation, hemodynamics will not be a stake until late in course. Timely surveillance combined with intervention can reduce morbidity and mortality.

#### Structural Valve Deterioration

SVD is changed to the function of a bioprosthesis from a valve abnormality. In fact, SVD is limited to bioprosthesis and absent in mechanical valves^[31]^. It consists of insufficient type or stenotic type, which is often characterised by calcification of the prosthesis. Valvular function deteriorates while heart function compensates. Prosthesis failure is the end result and requires reoperation. Thus, the incidence of SVD is constantly monitored in studies. This affects whether such prosthesis is suitable for young adults, who, due to longer life expectancy, will have higher lifetime chances of suffering from SVD.

Therefore, efforts should be devoted to reducing SVD. One question is whether different types of bioprosthesis affects the SVD rate. Mitroflow LXA valve has a SVD rate of 47% at 2 years and 82% at 3 years^[35]^; and Hancock II has a freedom from SVD of 29.2% at 20 years^[36]^. However, Mohammadi et al. studied 475 patients receiving Freestyle stentless bioprosthesis. The freedom rate from SVD reaches 95.8% at 10 years^[37]^. Bourguignon et al. reports the SVD rate in CEP valves in patients younger than 60 years old as 84% at 10 years^[25]^.

Another question is if SVD is a modifiable factor. In fact, the incidence of early stenosis-type SVD is shown to be related to patient-prosthesis mismatch (PPM)^[38]^. Interestingly, prosthesis size is to correlated to long-term survival and reoperation rate of SVD. Christ et al.^[26]^ claimed that, instead of age or prosthesis type, in patients with implant size ≤25 mm has a significant lower survival rate compared to patients with larger prostheses. As PPM can be a causative factor to stenosis-type SVD, the incidence of PPM can be minimised by increasing the aortic valve annulus during replacement surgery, through allowing the insertion of a larger size prosthesis. It seems worthwhile to explore whether surgical technique can reduce long-term reoperation from SVD by minimising PPM in young adults.

#### Anticoagulation

As discussed above, high bleeding rate in mechanical valves is attributed to the need for anticoagulation. However, warfarin administration affects patients in more ways. The protocol for anticoagulation in mechanical prosthesis is rigorous. Depending on the type of mechanical valve, the international normalized ratio (INR) target stands at 2.0-3.5 times of normal patients. The VA study examined high-level anticoagulation compared to low-level anticoagulation^[22]^. High dose anticoagulation brings some benefits of antithrombosis but more risk of bleeding^[39]^. Gastrointestinal bleeding can be a result.

Aside from risk of bleeding, anticoagulation also affects patients’ quality of life. Lifelong anticoagulation can cause inconvenience. Adherence can be an issue as younger adults tend to be less complacent with warfarin^[40]^. hindering the entire management in patients receiving mechanical prosthesis. As for patients suffering from atrial fibrillation, INR target is further increased by 0.5 times the normal^[32]^. Bioprosthetic valve patients with atrial fibrillation are also administered with anticoagulation with an INR targeting 2.0 to 2.5. It seems that in patients with atrial bioprosthetic valve with atrial fibrillation as a comorbidity, patients lose the advantage of bioprosthesis reducing bleeding incidences. However, atrial fibrillation is often age-related and young adults are less likely to suffer from it. They have a higher chance of enjoying freedom from anticoagulation compared to elderly patients. Therefore, bleeding events can be minimised.

#### Quality of Life

Ruel et al.^[32]^ also found that patients receiving bioprosthetic valves are more likely to enjoy a higher quality of life indicated by the higher score in the physical component of 12-Item Short-Form Health Survey (SF-12). Patients with bioprosthetic valves are more likely to be free from recurrent heart failure and disability, which have a significant influence on their quality of life. Added to all this, they have a better self-awareness – they are more satisfied with heart valve replacement and are less likely to perceive that heart valve replacement has impacted their daily lives.

#### Development in Bioprosthesis

With regard to developments in bioprosthesis for SAVR, there have been engagements in multiple fronts, including surgical approaches, incision method and valve design.

With the current bioprosthesis in use, surgical techniques are hoped to increase prosthesis longevity in young patients. According to Johnston et al.^[40]^, postoperative initial transvalvular gradient is related to prosthesis explantation for SVD and is more significant in young patients. It was also demonstrated that the small area of the valve orifice is related to a greater chance of stenosis-type SVD. Using selective root enlargement and choosing prostheses with larger orifice area carry the hopes of minimising SVD in the young with currently available prostheses. The sutureless valve is a new type of bioprosthesis. It is already mounted on the stent and requires minimal sutures during surgery, thus reducing bypass and cross-clamping times. High-risk patients susceptible to prolonged operation can benefit more from it^[15]^. Rubino et al.^[41]^ studied the outcome of 314 patients receiving Sorin Perceval S bioprosthesis, of which 86.3% had ejection fraction >50% and mean age in 77.9 ± 5 years. In-hospital mortality of isolated valve replacement was only 1.4%. This lays the foundation of applying surgical valve replacement to high-risk patients previously considered unsuitable for extensive surgeries. Population of other risk profiles may also benefit from it in the future.

### Treatment for High-Risk Aortic Stenosis Patients

#### The Generation of Valvuloplasty

The gold standard of severe AS treatment has been the surgical aortic valve replacement (SAVR). Nevertheless, advanced age, left ventricular dysfunction and other comorbidities make SAVR infeasible in at least 30% of patients with severe AS^[42]^. Prior to the prevalence of TAVR, patients with severe AS who are not surgical candidates would rely on medical treatment or balloon valvuloplasty. Median survival was only 2-3 years, on average, after symptoms appeared^[43,44]^.

##### Evidence of TAVR

Ever since percutaneous heart valve was implanted in humans in 2000 by Bonhoeffer et al.^[45]^ to pulmonary prosthetic conduits, transcatheter approach of heart valve replacement was constantly under the spotlight. The first successful aortic valve replacement performed with transcatheter approach was reported by Cribier et al.^[46]^ in 2002. Since then, transcatheter aortic valve replacement (TAVR) has provided new hope for inoperable high-risk patients. Gaining popularity, the number of TAVR performed in the last decade has exceeded 100,000^[46]^. Balloon valvuloplasty now serves as a bridge for patients who are hemodynamically unstable for both surgery and TAVR, awaiting definitive surgery. Clinical trials were conducted to compare the outcomes of TAVR with SAVR in different patient populations. Among them, Placement of Aortic Transcatheter Valve (PARTNER) was a large clinical study investigating the outcomes in patients with high risk in operation. The 358 patients involved were divided into two cohorts. In one cohort, patients were randomly assigned to receive either TAVR or SAVR. The 30-day mortality was 3.4% for TAVR and 6.5% for SAVR. At 1 year, the death rate of TAVR and SAVR were 24.1% and 26.8%, respectively, showing similar mortality in one year. However, they were associated with different morbidities. In the short-term, TAVR was associated with a higher rate of major stroke (30 days at 3.8% *vs*. 2.1%, *P*=0.20; 1 year at 5.1% *vs*. 2.4%, *P*=0.07) and more major vascular complications (11.0% *vs*. 3.2%, *P*<0.001). In contrast, less major bleeding (9.3% *vs*. 19.5%, *P*<001) and new-onset atrial fibrillation (8.6% *vs*. 16.0%, *P*=0.006) were noticed in the TAVR group^[47]^.

In another cohort, patients unsuitable for SAVR underwent TAVR or standard therapy, including balloon valvuloplasty. The 30-day mortality was 5.0% in the TAVR group and 2.8% in the standard therapy group, but the mortality rate from any cause was significantly lower in the TAVR group. TAVR was associated with more strokes at 30 days (5.0% *vs*. 1.1 %, *P*=0.06) and 1 year (7.8% *vs*. 3.9%, *P*=0.18)^[47]^. The advantage of TAVR in the treatment of high-risk AS patients over surgery or valvuloplasty was demonstrated.

Leon et al.^[42]^ further conducted the PARTNER 2 cohort, a randomised trial looking into TAVR in AS patients with intermediate risks. In terms of survival, 2-year mortality was similar in the TAVR group and in the surgery group (16.7% *vs*. 18%), with similar rates of disabling stroke (6.2% *vs*. 6.4%). In terms of complications, less major bleeding, acute kidney injury and atrial fibrillation were found in the TAVR group, while surgical patients had fewer vascular complications. PARTNER 2 findings are incongruent with the previous PARTNER Trial.

Adams et al.^[48]^ conducted another randomised trial on TAVR in which 995 patients with high risks of operation were randomly assigned to undergo TAVR or SAVR. Patients in the TAVR group received Corevalve, a self-expanding porcine pericardial valve. The 1-year mortality was 14.2% in TAVR and 19.1% in SAVR group. Main cardiovascular and cerebrovascular events (20.4% *vs*. 27.3%) and the rate of stroke (8.8% *vs*. 12.6%) were lower in the TAVR group. Even more patients in TAVR were complicated with main vascular events and require pacemaker implantations. In the third year, however, the absolute risk reduction of TAVR in terms of death from any cause or stroke was 9.4% when compared with SAVR^[48]^.

It comes to realisation that, within selected groups of high and even intermediate risk patients, TAVR is not inferior to SAVR in patients considering the survival rate. With the use of Corevalve, TAVR has shown a lower mortality rate when compared with SAVR in patients with high surgical risk. On the other hand, specific complications tend to occur more in different modalities requiring clinical attention: TAVR is more likely associated with major vascular complications, while SAVR comes with haemorrhage and atrial fibrillation.

The boundary between TAVR and SAVR indication is in the blurring. Shall transcatheter prosthesis continue to develop, it might as well be indicated in patients with lower surgical risks providing comparable outcomes as surgical approach in a larger group of patients.

##### Role of Medical Treatment

The role of pharmacological treatment is limited in the treatment of AS. It is generally accepted that valve replacement is offered to symptomatic patients. Pharmacological treatment is mainly offered to: 1) high-risk or inoperable patients; 2) stop the progression from asymptomatic to symptomatic AS.

As for patients who cannot undergo surgery, for reasons of poor morbidities or unfavourable anatomy, clinical treatment aims to normalise cardiovascular function and prevent superimposed conditions. Controlling hypertension and volume statue are fundamental to preserving cardiovascular function. For this purpose, diuretics, beta-blockers and vasodilators are prescribed.

As for controlling disease progression, there is no widely accepted or agreed regimen. Trials on efficacy of statins and angiotensin-converting-enzyme inhibitors have been carried out, but randomised trials show no effect^[49,50]^.

Yet for younger patients whose BAV is often an etiology of AS, pharmacological treatment plays a bigger role in their treatment. BAV is often associated to aortic dissection apart from valvular disease^[51]^. Employing beta-blocker might be able to provide a holistic treatment to this group of patients^[6]^. Further prospective studies are needed to look into how the pros and cons of beta-blockers are balanced. Another potential drug that may contribute to slow the progression of early AS is statins, as we know the process of atherosclerosis and calcification contributes to valvular calcification^[52]^. Some reports have claimed its beneficial effect^[53,54]^. However, randomised trials and systematic reviews have shown otherwise^[55]^. Meanwhile, it is known that modifying risk factors of atherosclerosis has benefits in slowing the AS progression. Thus, when it comes to AS in the young, it is more advisable that those risk factors be monitored as we await more evidence on medical treatment, as conjunct to the surgical treatment.

### Unanswered Clinical Questions

#### Concomitant CABG and AVR

Concomitant CABG performed with SAVR has been an independent risk factor for postoperative survival. Indication for this procedure includes: 1) patients with symptomatic aortic valve pathology with coronary heart disease discovered during preoperative angiogram; 2) patients requiring CABG with their underlying aortic valve pathology revealed in preoperative investigation; and 3) patients with concurrent aortic valve and coronary artery diseases. It is mostly agreed that CABG should be provided during SAVR in patients expected to have acceptable long-term survival rate. Multiple studies have reported a decrease in survival for combined surgery, compared to isolated SAVR. Alsoufi et al.^[56]^ have reported 249 patients undergoing concomitant CABG with SAVR, observing that long-term survival is compromised compared to isolated SAVR, or isolated CABG. The same conclusion is drawn by Ruel et al.^[32]^. This could be a result of the longer surgery time for a complex procedure, or the higher likelihood of patients with concurrent diseases carrying comorbidities.

However, the choice of prosthesis is still debated. Akins et al.^[57]^ performed a retrospective cohort on 750 patients who received combined SAVR and CABG procedures. They claimed that patients who receive bioprostheses had a better outcome, fewer related complications like myocardial infarction and the need of reintervention and lower cardiac mortality^[57]^. It can be attributed to mechanical valves a greater cardiac compromise or, conversely, the group of patients receiving bioprostheses are older than the mechanical group and are more likely to develop other life-threatening diseases. Contrarily, Alsoufi et al.^[56]^ reported that bioprosthetic valve patients receiving combined procedure are associated with greater early morbidity, compared to f mechanical prosthesis recipients. This difference may again be contributed by older age, poorer NYHA function, and other characteristics in patients that physicians tend to offer bioprostheses^[56]^.

The study remains inconclusive in terms of guiding choice of valve in concomitant AVR and CABG. When the age threshold of bioprosthesis implantation is also concerned in the context of combined procedures, further studies should be carried out to determine whether it will benefit younger patients with better baseline comorbidities. It can only be concluded that there is a wide variety of patients warranting the combined CABG and SAVR procedure. Efforts should be devoted to identifying the extent of coronary artery disease and aortic valve disease, comorbidities and other factors in order to provide a tailor-made treatment in the best interest of patients.

#### Coronary Artery Disease and TAVR

From 40 to 75% of patients undergoing TAVR have significant coronary artery disease (CAD), which can be managed by percutaneous coronary intervention (PCI)^[58]^. There has been debate on whether PCI should be done before, combined or after TAVR. Holistic evidence on the long-term outcomes of various approaches is still lacking.

Compared with the combined procedure, PCI prior to TAVR allows the patient to undergo a lower accumulated contrast dose. A study has shown that the contrast dose received by patients with PCI before TAVR was lower than that received by those receiving concomitant PCI and TAVR (292.3 ± 117.5 *vs*. 171.9 ± 68.4 ml)^[59]^. This is of particular importance because renal impairment is prevalent in patients receiving TAVR. Added to that, conducting PCI before TAVR may reduce the chance of hemodynamic instability in the TAVR procedure^[60]^. However, this result has not been reproduced by other studies.

For concomitant PCI and TAVR procedure, there are advantages and disadvantages shown in different studies. Penkalla et al. reported comparable survival in up to 3 years in CAD patients having TAVR and PCI and patients without CAD having TAVR alone^[61]^. However, Singh et al. showed that patients receiving concomitant PCI and TAVR have significantly higher mortality when compared with patients receiving TAVR alone (10.7% *vs*. 4.6%)^[62]^. More studies are needed to comprehend the long-term outcomes of concomitant PCI and TAVR on patients.

Concerning PCI after TAVR, there are only a few reports reporting its outcome, probably as a result of a major technical problem – cardiologists may experience difficulty accessing the coronary ostia for PCI after TAVR^[60]^. Its long-term effect still needs to be confirmed.

#### Minimally Invasive SAVR

In the recent decade, the minimally invasive approach to aortic valve replacement has become increasingly popular. By definition, it aims to achieve access to the aortic valve without full sternotomy. It can be classified into intercostal access and limited sternotomy^[63]^. It aims to cause less pain to patients, achieving faster recovery, shorter hospitalisation, less blood loss and less trauma. Recently, Nguyuen et al^[64]^. have published a multi-institutional retrospective review of 1,503 patients receiving minimally invasive SAVR. Among patients with ejection fraction >40%, it was found that they have better short-term outcome – including less bleeding, shorter ICU stay and hospitalisation and new-onset atrial fibrillation than AVR patients with full sternotomy, while in patients with EF <40% the difference is not significant. However, it was found that the minimally invasive approach would need a longer CPB and cross-clamping time^[65]^. The procedure becomes more challenging and would be a risk factor for high-risk patients. In younger patients, however, it might be a considerable alternative, as they are more likely to be able to endure a longer procedure^[66]^. Combined with the sutureless prosthesis, faster implantation can translate into reduced operative time in the minimally invasive approach^[67,70]^. It does not only apply to younger but higher risk population, especially the population group indicated with TAVR.

## CONCLUSION

The choice of aortic valve prosthesis in young patients with AS is far from a straightforward one. Possessing the advantages of freedom from lifelong anticoagulation, fewer prosthesis complications and patient perception, together with comparable long-term survival and modest reoperation risk, bioprosthesis does have the edge over mechanical prosthesis in certain aspects. Concerns in SVD, although its odds are modifiable, cannot be omitted. Despite multiple studies demonstrated excellent long-term outcomes in various types of bioprosthesis and advocate the reduction of the age threshold of bioprosthesis implantation, literature concerning the long-term performance in younger individuals under 50 is still insufficient to be conclusive. On the other hand, developments in the management of aortic valve pathologies are seen in all fronts, from surgical approach to TAVR. Choice of treatment has become complex and multifactorial. Decisions that a heart team have to worry about become more difficult. However, there is no doubt that the long-term benefits of patients can be anticipated.

**Table:** 

Authors’ roles & responsibilities	
AH	Substantial contributions to the conception or design of the work; or the acquisition, analysis, or interpretation of data for the work; final approval of the version to be published
MMYS	Substantial contributions to the conception or design of the work; or the acquisition, analysis, or interpretation of data for the work; final approval of the version to be published
CHMW	Substantial contributions to the conception or design of the work; or the acquisition, analysis, or interpretation of data for the work; final approval of the version to be published
ARM	Substantial contributions to the conception or design of the work; or the acquisition, analysis, or interpretation of data for the work; final approval of the version to be published
MB	Substantial contributions to the conception or design of the work; or the acquisition, analysis, or interpretation of data for the work; final approval of the version to be published
